# The Decline of Deep Brain Stimulation for Obsessive–Compulsive Disorder Following FDA Humanitarian Device Exemption Approval

**DOI:** 10.3389/fsurg.2021.642503

**Published:** 2021-03-12

**Authors:** Heather Pinckard-Dover, Herbert Ward, Kelly D. Foote

**Affiliations:** ^1^Department of Neurosurgery, University of Florida, Gainesville, FL, United States; ^2^Norman Fixel Institute for Neurological Diseases, University of Florida Health, Gainesville, FL, United States; ^3^Department of Psychiatry, University of Florida, Gainesville, FL, United States

**Keywords:** deep brain stimulation, obsessive—compulsive disorder, humanitarian device exemption (HDE), healthcare disparities, YBOCS = Yale-Brown Obsessive Compulsive Scale

## Abstract

**Background:** In February 2009, the US Food and Drug Administration (FDA) granted Humanitarian Device Exemption (HDE) for deep brain stimulation (DBS) in the anterior limb of the internal capsule (ALIC) for the treatment of severely debilitating, treatment refractory obsessive–compulsive disorder (OCD). Despite its promise as a life altering treatment for patients with otherwise refractory, severely debilitating OCD, the use of DBS for the treatment of OCD has diminished since the FDA HDE endorsement and is now rarely performed even at busy referral centers. We sought to identify factors hindering OCD patients from receiving DBS therapy.

**Materials and Methods:** University of Florida (UF) clinical research databases were queried to identify patients evaluated as potential candidates for OCD DBS from January 1, 2002 to July 30, 2020. A retrospective review of these patients' medical records was performed to obtain demographic information, data related to their OCD, and details relevant to payment such as third-party payer, study participation, evaluation prior to or after HDE approval, and any stated factors prohibiting surgical intervention.

**Results:** Out of 25 patients with severe OCD identified as candidates for DBS surgery during the past 18 years, 15 underwent surgery. Prior to FDA HDE approval, 6 out of 7 identified candidates were treated. After the HDE, only 9 out of 18 identified candidates were treated. Seven of the 9 were funded by Medicare, 1 paid out of pocket, and 1 had “pre-authorization” from her private insurer who ultimately refused to pay after the procedure. Among the 10 identified OCD DBS candidates who were ultimately not treated, 7 patients—all with private health insurance—were approved for surgery by the interdisciplinary team but were unable to proceed with surgery due to lack of insurance coverage, 1 decided against surgical intervention, 1 was excluded due to medical comorbidities and excessive perceived surgical risk, and no clear reason was identified for 1 patient evaluated in 2004 during our initial NIH OCD DBS trial.

**Conclusion:** Based on compelling evidence that DBS provides substantial improvement of OCD symptoms and markedly improved functional capacity in 2 out of 3 patients with severely debilitating, treatment refractory OCD, the FDA approved this procedure under a Humanitarian Device Exemption in 2009, offering new hope to this unfortunate patient population. A careful review of our experience with OCD DBS at the University of Florida shows that since the HDE approval, only 50% of the severe OCD patients (9 of 18) identified as candidates for this potentially life altering treatment have been able to access the therapy. We found the most common limiting factor to be failure of private insurance policies to cover DBS for OCD, despite readily covering DBS for Parkinson's disease, essential tremor, and even dystonia—another HDE approved indication for DBS. We have identified an inherent discrimination in the US healthcare system against patients with medication-refractory OCD who are economically challenged and do not qualify for Medicare. We urge policy makers, insurance companies, and hospital administrations to recognize this health care disparity and seek to rectify it.

## Introduction

In February 2009, the US Food Drug Administration (FDA) approved the use of Medtronic “Reclaim” bilateral anterior limb of internal capsule (ALIC) deep brain stimulation (DBS) (Medtronic, Minneapolis, MN) for the treatment of chronic, severe, treatment-resistant obsessive compulsive disorder (OCD) in adult patients who have failed at least three selective serotonin reuptake inhibitors (SSRIs) under an humanitarian device exemption (HDE) (1). This approval came after reviewing the preliminary data of multiple prospective trials dating as far back as 1999 showing responder rates from 33 to 78% (“responder” ≥35% reduction in Yale-Brown Obsessive–Compulsive Scale (YBOCS) scores) (2–6). These results compare favorably to ablative therapies that have been practiced for many years (7). The initial OCD DBS studies intended to recapitulate the targets ablated during classic capsulotomy procedures, targeting the entirety of the anterior limb of the internal capsule (ALIC). With ongoing clinical experience, it was determined that the majority of the clinical benefit was achieved through more ventral and posterior stimulation within the ALIC, and the target was refined to focus on the ventral ALIC and the nucleus accumbens (NA)—now commonly referred to as the ventral capsule/ventral striatum (VC/VS) (8). Over the years, other targets such as the nucleus accumbens (NA) (9–13), subthalamic nucleus (STN) (12, 14, 15), bed nucleus of stria terminalis (BNST) (9, 10, 16), inferior thalamic peduncle (ITP) (17, 18), thalamus (19), medial forebrain bundle (MFB) (20), caudate (12), and anteromedial globus pallidus internus (amGPi) (21) have been proposed as potential targets for OCD DBS. Long term data shows continued benefit for many of the initial responders (22–32). As one of the early centers to build on the pioneering work of Bart Nuttin and his team in Belgium (2, 3) and Ben Greenberg et al. in the US (5), our team at UF was the first to perform an NIH-sponsored trial of DBS for OCD (launched in 2002) (6) and one of the centers providing early evidence in support of the ultimate HDE approval. We experienced first-hand the frequently dramatic, and intensely gratifying improvement in symptoms and quality of life that this therapy commonly provides to desperate patients with severely debilitating OCD and to their families. Despite our experienced team, a very high-volume clinical DBS program, and unrelenting efforts to provide DBS therapy to appropriately selected patients with severe OCD, we have managed to perform only a few OCD DBS procedures at our center since the HDE approval in 2009. We sought to identify factors that are limiting patient access to this potentially life altering therapy.

## Materials and Methods

After Institutional Review Board (IRB) approval, queries of the University of Florida INFORM clinical research database and our Integrated Data Repository (IDR) were performed to identify patients who met criteria for OCD DBS therapy as follows: age over 18 years, ICD code for OCD (300.3, F42.9, F42.8, F42.2, F42.1, F42.0, or F42), Yale-Brown Obsessive–Compulsive Scale (YBOCS) score greater than 28, and evaluation for DBS by our primary psychiatrist (HW) and either primary neurosurgeon (KF) or our primary neuropsychologist (Dawn Bowers) between January 1, 2002 and July 30, 2020. During chart review, to narrow the evaluation to only patients deemed appropriate candidates for OCD DBS therapy by the psychiatrist “gatekeeper,” patients seen only by the primary psychiatrist (HW) with no referral to remaining members of the interdisciplinary board for evaluation as a candidate for DBS therapy, were excluded, as were those presented to the interdisciplinary board as candidates for DBS to treat disorders other than OCD. A retrospective review of the medical record of each of the patients identified by this query was performed to gather the patient's demographic information (age, sex, comorbidities, and disease duration), pre- and post-operative YBOCS score, payer source, participation in either our initial single-center or subsequent multi-center NIH OCD DBS trial, timing of surgery relative to the 2009 HDE (before or after), and any factors in the record reported to prevent a patient from proceeding with surgical intervention.

## Results

Query of our databases identified 28 patients who were appropriate OCD DBS candidates based on the stated inclusion criteria. Retrospective chart review resulted in elimination of 3 patients with (comorbid) OCD who were evaluated by the primary psychiatrist and the primary neurosurgeon as candidates for DBS to treat disorders other than OCD (essential tremor, Parkinson's Disease, and Tourette's Syndrome).

Out of 25 patients with severe OCD identified as appropriate candidates for DBS surgery during the past 18 years, 15 underwent surgery. Prior to FDA HDE approval, 6 out of 7 identified candidates (86%) were treated (all funded by our NIH grant). After the HDE, only 9 out of 18 identified candidates (50%) were treated. Six of the 9 post HDE patients treated surgically were funded by Medicare, 1 patient who underwent surgery had Medicaid, but his coverage was denied post-operatively and the procedure was written off by the hospital, 1 wealthy patient with a private insurance policy that denied coverage paid out of pocket, and 1 patient with private insurance obtained “pre-authorization” for the surgery after several appeals to her insurer, underwent DBS surgery in 2011, only to have payment ultimately denied by the insurer (“bait and switch”) (33).

Among the 10 identified OCD DBS candidates who were ultimately not treated, 7 patients—all with private health insurance—were approved for surgery by the interdisciplinary board, but were unable to proceed with surgery due to lack of insurance coverage for this specific procedure by their insurer, 1 patient decided the risk of DBS surgery was unacceptable and opted to try transcranial magnetic stimulation (TMS), 1 was excluded by the interdisciplinary DBS board because of excessive perceived surgical risk due to significant medical comorbidities, and no clear reason was identified for 1 patient from 2004 during our initial NIH OCD DBS trial.

OCD DBS responders are defined as patients who have at least a 35% reduction in their YBOCS score at 1 year. Of the 15 OCD patients implanted at the University of Florida, pre-operative and 1-year post-operative YBOCS scores were available via retrospective chart review for 11 patients. Seven (63.64%) of these patients were responders at 1 year. The average age of those patients who underwent surgery was 49 vs. 40 in those candidates who did not proceed to surgery. There were 6 males in the surgical group and 3 in the non-surgical group. The average disease duration was 40 years in the surgical group and 16.11 years in the non-surgical group. Average pre-operative YBOCS score for the surgical group was 35 and 32 in the non-surgical group. Co-morbidities were similar between groups and are listed in Table 1. In the non-surgical group, one patient was deemed an inappropriate candidate for surgical intervention due to severe interstitial cystitis that interfered with her ability to interact outside the home even more than her OCD.

**Table 1:** OCD patient demographics.

	**Surgery**	**No surgery**
Age (years)	49.11	40.22
Sex (M:F)	6:3	4:5
Disease duration (years)	40	16.11
Initial YBOCS	35.14	31.71
Co-morbidities	GAD, MDD, DM, OSA, GERD, HTN, BCC, SCCa,	IC*, GAD, MDD, substance abuse, bulimia, HTN, GERD, PCOS, hypothyroidism, POTS, neuromuscular disorder
payer source	6 Medicare 1 Medicaid 2 Private Insurance (1 Self Pay, 1 bait and switch)	1 Medicare 8 Private Insurance

*YBOCS, Yale-Brown Obsessive–Compulsive Scale; IC, Interstitial Cystitis; GAD, General Anxiety Disorder; MDD, Major Depressive Disorder; DM, Diabetes Mellitus; OSA, Obstructive Sleep Apnea; GERD, Gastro-Esophageal Reflux Disorder; HTN, Hypertension; BCC, Basal Cell Carcinoma; SCCa, Squamous Cell Carcinoma; PCOS, Poly-Cystic Ovarian Syndrome; POTS, Postural Orthostatic Tachycardia Syndrome*.

* *The only comorbidity that prohibited surgical intervention*.

## Discussion

When a novel therapeutic intervention is developed for the treatment of patients with a rare disorder, it is difficult to gather enough clinical evidence to meet the FDA standard of reasonable assurance of safety and effectiveness. Not only is it impractical to perform a large prospective randomized trial of the intervention due to insufficient numbers of cases, but the small prospective market for any therapeutic device involved represents a strong disincentive for those who might invest in the development of such therapies. In order to address these challenges that disadvantage patients with rare disorders, Congress included a provision in the Safe Medical Devices Act of 1990 to create a new regulatory pathway for products intended for diseases or conditions that affect small (rare) populations—the Humanitarian Device Exemption (HDE) Program (34).

Humanitarian Device Exemptions were created to improve access to therapies for patients with rare disorders. While OCD is not an uncommon disorder, the number of people with severely debilitating, medication refractory OCD who would be appropriate candidates for DBS therapy is certainly well below 8,000 per year in the US (the legally defined threshold for HDE qualification). The quality of life of patients afflicted with such treatment refractory, severe cases of OCD is abysmally poor, but two out of three of these severely afflicted patients respond to DBS. The therapeutic benefit we have observed in most patients is typically dramatic, restoring function and quality of life to a degree that has been extremely gratifying. Those of us involved in formal studies were encouraged when the HDE was granted, expecting that third party payers would now cover the cost of the operations and we would no longer need to include that cost in our NIH budgets. Unfortunately, this expectation was not met. Insurers refused to pay for the procedure despite the HDE, and our large multicenter OCD DBS trial essentially died due to inability to recruit subjects under the third-party payer model. The genesis of the current project stems from our frustration with the observation that we are performing far fewer OCD DBS cases per year since the HDE than we did prior to its approval. The main goal of the HDE is improved access, but we have demonstrated with this analysis that access to OCD DBS has actually diminished in our cohort of patients with medication refractory, severely debilitating obsessive–compulsive disorder since the HDE was granted.

The HDE mechanism requires that each institution performing the procedure covered by the HDE have local IRB approval and follow a minimum protocol for quality assurance and the gathering of some standardized outcomes data regarding the effects of the HDE approved intervention. Realistically, centers with higher volume and experience with clinical research tend to be best equipped to manage these protocols and maintain compliance with an FDA HDE. By design, the overhead required to maintain approval and compliance with an HDE tends to discourage smaller, less experienced centers, and has the desirable effect of steering patients with rare disorders to larger regional referral centers. Although it is markedly inferior evidence to that generated by a prospective trial, the HDE, if protocols are followed, can encourage the generation of some useful, shareable data on outcomes of the intervention in question. Many centers are working together to review combined data and determine predictive factors for patient response to therapy (35–40) and possible mechanisms of action (38, 41–49). The more data available for review, the more we can understand these mechanisms and tailor the therapy to the needs of individual patients to improve outcomes.

With an HDE in place designed to improve access, the question becomes, “Why are so few patients with severely debilitating, medication refractory OCD, actually getting the treatment that could provide them such important benefit?” This review sought to identify common limiting factors. Only half of the patients deemed candidates for OCD DBS at our center since the HDE were treated. Because of policies widely adopted by private insurers, access to the procedure appears to be essentially limited to patients with Medicare, or those with uncommon wealth sufficient to pay out of pocket. Medicare coverage is limited to US citizens >65 years of age (not the typical age group of these patients), or those collecting Social Security Disability Insurance (SSDI) insurance for a minimum of 24 months. Qualifying for SSDI for psychiatric disorders is challenging, and requires extensive medical documentation of symptoms, treatment, and inability to function outside the home or inability to work for a minimum of 12 months. OCD patients who are appropriate candidates for DBS should generally qualify for SSDI, but to do so they must not work for 12 months, then be on disability for 24 months prior to qualifying for Medicare coverage for DBS. This represents a typical delay in treatment of over 3 years for patients with extraordinarily poor quality of life.

If a patient does not have, or cannot qualify for, Medicare coverage, they have two remaining options: pay out of pocket or travel to the nearest center conducting a fully funded clinical trial. Currently there are only six open trials of DBS for OCD according to ClinicalTrials.gov, and most presumably do not cover the cost of the procedure. Even if the cost of the procedure were covered, extensive travel is both logistically and financially prohibitive for most patients with severely debilitating OCD. Our one self-pay patient was required to pay $104,000 out of pocket prior to scheduling her surgery. Such out-of-pocket expenses are unobtainable for the vast majority of Americans, let alone those encumbered with burden of debilitating OCD. Based on our analysis, it appears that access to this potentially life altering therapy is extremely limited for the non-wealthy—an example of the far too common healthcare disparity in our current healthcare system.

How then do we correct this discrimination against the non-wealthy and avoid the prolonged suffering and treatment delays? Short of radical health care reform, the solution appears to be to persuade private insurers to cover DBS for OCD. We have tried traditional methods of appealing insurer's denial with evidence by published quantitative trials, cost-effectiveness trials, and peer-to-peer reviews with little avail. We are hopeful that highlighting the problem with publications like this one will have some persuasive effect. Helping policy makers to understand the terrible plight of these patients and the dramatically beneficial effect the therapy provides to most patients would presumably have a positive effect. Though citation of quantitative data, such as the fact that two thirds of otherwise treatment refractory patients respond to the treatment with >35% reduction in YBOCS scores, is helpful, sharing qualitative data describing patients' experiences may more powerfully illustrate the true benefit of the therapy. A few studies provide such qualitative data (25, 50–52), but are not published in high impact journals and probably have not been viewed by many insurance company executives or their medical consultants. Media attention to the issue might help advance the cause as might the publication of more cost-effectiveness studies such as those by Moon and Ooms (53, 54). These studies were carried out in Korea, United Kingdom (UK), and the Netherlands, but none have been carried out in the United States. Moon et al. used a Markov model to estimate cost-effectiveness of DBS vs. traditional therapy over a 10 year horizon and report a ratio based on quality-adjusted life-year (QALY). They found a cost-effectiveness ratio of $37,865/QALY in Korea and $34,462/QALY in the UK. Ooms et al. used a 2-year prospective model and found €65,394 gain per QALY when using a rechargeable generator as is recommended in this subgroup given their high frequency use. These are substantial cost savings that could also be relayed to the insurers if they chose to evaluate long term benefits.

Though this review of our experience clearly shows that non-Medicare insurance is the most important factor limiting access to OCD DBS among patients deemed appropriate candidates for this therapy at our center, there are limitations that should be considered when interpreting our results. The cohort studied here is small with only 25 patients reviewed. Despite best efforts, the retrospective nature of the study leaves some incomplete data including the inability to determine the limiting factor in 1 untreated patient. Our inclusion criteria required the patient be evaluated by both the primary psychiatrist (HW) and another key member of the interdisciplinary board as a surrogate marker of being deemed an appropriate candidate for OCD DBS therapy because there was not a direct order that was trackable in the database. Through discussions with the primary psychiatrist, we learned that after several patients were unable to access the surgery due to poor insurance coverage, he began to routinely discuss this potential problem with patients prior to referring them for further evaluation by the interdisciplinary DBS board. After this discussion, some indeterminate number of patients opted not to proceed with interdisciplinary evaluation for DBS knowing they could not afford the therapy. We can therefore assume that our methodology in this study underestimates the problem, and the percentage of appropriate OCD DBS candidates who are ultimately denied access to the therapy is even greater than documented here.

## Conclusion

DBS is an effective, often life-altering therapy for most appropriately-selected patients with severely debilitating, treatment refractory OCD, and appears to remain effective in limited long-term studies. Initial effectiveness studies provided sufficient evidence to persuade FDA reviewers to grant HDE approval in 2009 for use of Medtronic “Reclaim” bilateral anterior limb of internal capsule (ALIC) deep brain stimulation for the treatment of chronic, severe, treatment-resistant OCD in adult patients who have failed at least three SSRIs. Despite HDE approval, only 9 of 18 patients deemed appropriate candidates for OCD DBS therapy at the University of Florida since 2009 have undergone surgical intervention. This review identified inability to pay due to non-Medicare insurance coverage (private insurers refuse to pay) as the most common factor limiting access to the procedure. Our findings here provide a poignant example of the unfortunate disparities in access to quality care in the US healthcare system, which tends to discriminate against those with psychiatric disorders, and against the non-wealthy. Patients afflicted with severely debilitating OCD generally fall into both of these categories, and typically do not qualify for Medicare coverage. We urge policy makers, insurance companies, and hospital administrations to recognize this health care disparity and seek to rectify it.

## Data Availability Statement

The original contributions presented in the study are included in the article/Supplementary Material, further inquiries can be directed to the corresponding author/s.

## Ethics Statement

The studies involving human participants were reviewed and approved by University of Florida Institutional Review Board. Written informed consent for participation was not required for this study in accordance with the national legislation and the institutional requirements.

## Author Contributions

HP-D and KF conceived the project idea. HP-D collected the data and wrote the draft. HW and KF were the treating physicians and edited the paper. All authors contributed to the article and approved the submitted version.

## Conflict of Interest

The authors declare that the research was conducted in the absence of any commercial or financial relationships that could be construed as a potential conflict of interest.
