# Supplementary material for: The Decline of Deep Brain Stimulation for Obsessive–Compulsive Disorder Following FDA Humanitarian Device Exemption Approval
Source: Front Surg. 2021 Mar 12;8:642503. doi: 10.3389/fsurg.2021.642503 (PMC7994854; doi:10.3389/fsurg.2021.642503)
Supplement: Supplementary file 1 [file Table_1.DOCX]

| Patient | Age | Sex | Disease Duration | Co-morbidities | Initial YBOCS | 1 yr Post-op YBOCS | Payor Source | Initial NIH Study | 2nd NIH Study | Post-HDE | Surgery year | Year Evaluated | Reason for No Surgery |
| --- | --- | --- | --- | --- | --- | --- | --- | --- | --- | --- | --- | --- | --- |
| 1 | 32 | F | 20 | MDD | 38 | 22 |  | yes |  |  | 2003 |  |  |
| 2 | 52 | M | 16 | MDD, HTN, hyperlipidemia | 34 | 29 |  | yes |  |  | 2003 |  |  |
| 3 | 40 | M | 11 | MDD, GAD | 33 | 7 |  | yes |  |  | 2004 |  |  |
| 4 | 33 | M | 29 | TS, asthma, GERD | 34 | 3 |  | yes |  |  | 2004 |  |  |
| 5 | 32 | F |  | MDD, GAD | 32 | 29 |  | yes |  |  | 2005 |  |  |
| 6 | 27 | F | 22 | endometriosis, GERD | 34 | 3 |  | yes |  |  | 2007 |  |  |
| 7 | 50 | M | 32 | MDD | 33 | 16 | Medicare |  | yes | yes | 2009 |  |  |
| 8 | 71 | M |  | MDD |  |  | Medicare |  |  | yes | 2010 |  |  |
| 9 | 41 | M | 11 |  | 34 | 8 | Medicare |  | yes | yes | 2011 |  |  |
| 10 | 30 | M | 15 |  | 40 | 32 | Medicaid |  |  | yes | 2011 |  |  |
| 11 | 59 | F | 47 | MDD | 34 | 9 | Private |  | yes | yes | 2011 |  |  |
| 12 | 56 | F |  | MDD, binge eating, OSA | 36 | 34 | Medicare |  | yes | yes | 2013 |  |  |
| 13 | 60 | F | 30 | MDD, GAD, actinic keatosis, HTN, BCC, SCCa | | | Self Pay |  |  | yes | 2014 |  |  |
| 14 | 40 | M | 12 | MDD, GAD, DM, GERD | 34 |  | Medicare |  |  | yes | 2016 |  |  |
| 15 | 35 | M | 22 | GERD | 35 |  | Medicare |  |  | yes | 2017 |  |  |
| 16 | 42 | F |  |  |  |  |  |  |  |  |  | 2004 | Unclear |
| 17 | 50 | F | 15 | MDD, HTN, hyperthyroidism | 34 |  | Private |  |  |  |  | 2013 | Lack of coverage |
| 18 | 33 | F | 8 | MDD |  |  | Private |  |  |  |  | 2013 | Lack of coverage |
| 19 | 64 | F |  |  | 31 |  | Medicare |  |  |  |  | 2015 | Patient choice |
| 20 | 39 | M | 14 | MDD, GAD | 36 |  | Private |  |  |  |  | 2015 | Lack of coverage |
| 21 | 28 | F | 22 | GAD, hypothyroidism, PCOS | 31 |  | Private |  |  |  |  | 2015 | Lack of coverage |
| 22 | 30 | F | 6 | MDD, GAD, NMD, POTS, IC | 32 |  | Private |  |  |  |  | 2016 | Co-morbidities |
| 23 | 58 | M | 2 | MDD, HTN, GERD | 27 |  | Private |  |  |  |  | 2018 | Lack of coverage |
| 24 | 22 | M | 4 |  |  |  | Private |  |  |  |  | 2018 | Lack of coverage |
| 25 | 38 | M | 30 | GAD, substance abuse, bulemia | 31 |  | Private |  |  |  |  | 2019 | Lack of coverage |
